
JOURNAL INFORMATION
==============================
NLM Title Abbreviation: Open Forum Infect Dis
Journal ID: Open Forum Infect Dis
NLM Journal ID: 101637045
Journal ID: ofid

Open Forum Infectious Diseases

EISSN: 2328-8957
Publisher: Oxford University Press

ARTICLE INFORMATION
==============================
PMCID: PMC10473189
PMID: 37662452
DOI: 10.1093/ofid/ofad447
Article ID: ofad447
Article version: 1
Subjects: Insight Article, AcademicSubjects/MED00290

Role of the microbiome in essential metabolism in the human gut and its implications for Clostridioides difficile infection

Chopra Teena Detroit Medical Center and Vibra Hospital, Detroit, MI

Hylemon Phil Virginia Commonwealth University, Richmond, VA

https://orcid.org/0000-0002-2221-008X Tillotson Glenn GST Micro LLC, North, VA

Dr. Teena Chopra Detroit Medical Center and Vibra Hospital, Detroit MI – tchopra@med.wayne.edu
Publication date: 2023 Sep
Electronic publication date: 2023 Sep 1
Volume: 10
Issue: 9
Electronic Location ID: ofad447
2023 Sep 1
Copyright: © The Author(s) 2023. Published by Oxford University Press for the Infectious Diseases Society of America.
Copyright year: 2023
License: This is an Open Access article distributed under the terms of the Creative Commons Attribution License (https://creativecommons.org/licenses/by/4.0/), which permits unrestricted reuse, distribution, and reproduction in any medium, provided the original work is properly cited.
License URL: https://creativecommons.org/licenses/by/4.0/

Keywords: Clostridioides difficile, microbiome, metabolism, short chain fatty acids, bile acids
Page count: 6

==============================

Open Forum Infectious Diseases

Open Forum Infectious Diseases 2023; 10: 9
doi: 10.1093/ofid/ofad447

InSight

### Role of the microbiome in essential metabolism in the human gut and its implications for *Clostridioides difficile* infection

Teena Chopra [1], Phil Hylemon [2], Glenn Tillotson [3]

[1] Detroit Medical Center and Vibra Hospital, Detroit, MI
[2] Virginia Commonwealth University, Richmond, VA
[3] GST Micro LLC, North, VA

**Keywords:** *Clostridioides difficile*, microbiome, metabolism, short chain fatty acids, bile acids.

### The gut microbiome

The term gut microbiome describes the diverse group of microorganisms (microbiota) including bacteria, archaea, fungi, algae, and small protists that share the human intestinal space, as well as their genomes and environment [1]. The gut bacterial microbiota in most healthy individuals primarily comprise a diversity of Bacteroidetes (*Bacteroides* and *Prevotella* spp.) and Firmicutes (*Lactobacillus*, *Bacillus*, *Clostridium*, and *Ruminococcus* spp.) phyla, with a lower abundance of Proteobacteria and Actinobacteria [2].

### Functions of the gut microbiota and colonization resistance

A rich and diverse gut microbiome provides resistance to colonization of the gut by exogenous organisms and prevents expansion of potential pathogenic organisms within the gut through a variety of mechanisms [2, 3]:

- Competition for key nutrients
- Production of inhibitory bile acids
- Production of short-chain fatty acids
- Lowering the luminal pH
- Production of bacteriocins
- Maintenance and enhancement of intestinal barrier function
- Host immune modulation

### Dysbiosis in *Clostridioides difficile* infection

Dysbiosis is a perturbation, usually due to antibiotic treatment, in the microbiota composition, abundance, and/or diversity, which can have significant impacts on the functionality of the gut microbiome. One consequence of dysbiosis is the loss of colonization resistance to pathogens [2], such as *Clostridioides difficile*, a spore-forming,- Gram-positive, obligate anaerobic bacterium. Patients with *C. difficile* infection (CDI) typically have reduced richness and diversity in the gut microbiome [4-6]] and perturbed microbiota composition, in particular decreased members from the *Clostridia* and *Bacteroidia* classes and increased members from the *Gammaproteobacteria* and *Bacilli* classes, most commonly including *Enterobacterales* and *Enterococcacea* families [7-11]. These perturbations are frequently worsened by treatment with standard-of-care prescription CDI antibiotics. Consequences of this dysbiosis include metabolomic changes that are more favorable for mucosa-adhered *C. difficile* spores to germinate to their active vegetative form, which can produce toxins in the colon that disrupt the intestinal epithelial barrier and result in a host inflammatory response [12-14]. Among the metabolites implicated in this cascade of CDI infection are bile acids and short-chain fatty acids (SCFA).

### Bile acids

Bile acids were initially thought to be only involved in lipid digestion in the intestinal lumen and cholesterol solubilization in the bile but have now seem to have a range of other effects: contributing to the homeostasis of lipids, glucose and other metabolic substrate, in addition to affecting immune system functions as well as gut microbiome composition [15]. Modification of bile acids is a

© The Author(s) 2023. Published by Oxford University Press for the Infectious Diseases Society of America. This is an Open Access article distributed under the terms of the Creative Commons Attribution License (https://creativecommons.org/licenses/by/4.0/), which permits unrestricted reuse, distribution, and reproduction in any medium, provided the original work is properly cited.

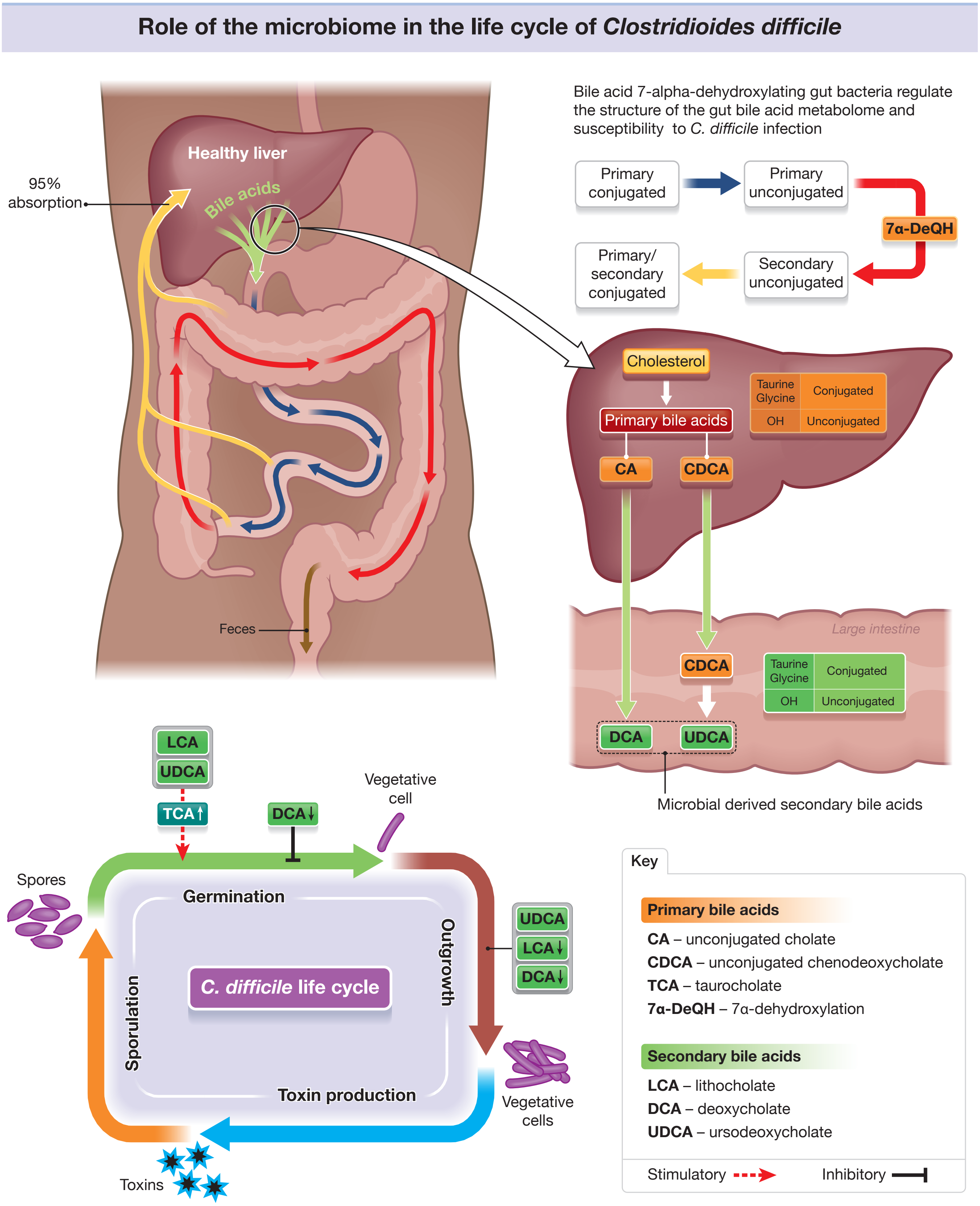

known mechanism by which gut microbiota provide colonization resistance to *C. difficile*. Liver-produced primary bile acids were initially thought to be only involved in lipid digestion in the intestinal lumen and cholesterol solubilization in the bile, but have now been shown to have a range of other effects: contributing to the homeostasis of lipids, glucose and other metabolic substrate, in addition to affecting immune system functions as well as gut microbiome composition [15]. Bile acids are excreted to the upper digestive tract as glycine or taurine conjugates (bile salts), most of which are reabsorbed in the distal ileum. Most of the primary bile salts that reach the colon are metabolized to secondary bile acids by commensal gut microbiota, which helps resist *C. difficile* colonization because primary bile acids, especially taurocholic acid, can stimulate spore germination via CspC pseudoprotease binding. It has been reported that some secondary bile acids inhibit spore germination. Further, several secondary bile acids may have a role in inhibiting *C. difficile* growth and/or toxin activity [16-22].

The primary-to-secondary bile acid conversion has been demonstrated to be important in CDI, as patients with CDI have lower secondary bile acids compared with healthy controls and higher primary bile acids in patients with recurrence versus those experiencing a first episode [20, 23]. Additionally, recovery from CDI seems to be correlated with recovery of secondary bile acids with multiple secondary bile acids in the large intestine shown to inhibit *C. difficile* spore germination and growth at physiological concentrations [24, 25].

### Short chain fatty acids

SCFAs are produced through bacterial fermentation of indigestible carbohydrates or dietary fiber. SCFAs are an important energy source for intestinal epithelial cells and play a vital role in maintaining epithelial function and colonization resistance. Three microbial-derived SCFAs in particular (butyrate, acetate, and propionate) have been associated with CDI resistance [17, 26, 27].

Butyrate, the main energy source for the colonic cells to proliferate and maintain the gut barrier, is metabolized through B-oxidation, and helps maintain an anaerobic milieu inside the colon [16]. It enhances the host innate antimicrobial immunity and cytokine-mediated immune response, maintains intestinal epithelial integrity, increases the expression of tight junction proteins, prevents epithelial damage by *C. difficile* toxins, reduces intestinal permeability, and inhibits intestinal inflammation and bacterial translocation [26, 28-30]. Acetate, enhances host immune function, stimulates immune cells to secrete anti-inflammatory cytokines, and mitigates the toxicity of *C. difficile* toxin A to colonocytes to ameliorate CDI [30, 31]. Finally, propionate promotes intestinal epithelial integrity and barrier function while reducing inflammation and oxidative stress [32-34].

The type and quantity of intestinal bacteria are the key factors determining the type and concentration of SCFAs. Therefore, one manifestation of intestinal dysbiosis in CDI patients is the decreased abundance of SCFA-producing bacteria, as the Firmicutes phyla produces butyrate (*Eubacterium rectale*, *Roseburia inulinivorans*, *Faecalibacterium prausnitzii*) whereas the Bacteroidetes phyla produce acetate (*Blautia obeum*) and propionate (*Bacteroides uniformis*, *Bacteroides vulgatus*) [30]. Depletion of these taxa are strongly associated with respective SCFA derangement. [35, 36].

### The antibiotic conundrum in the treatment of CDI

Antibiotic therapy is the standard course of treatment for rCDI [37], however, this represents a therapeutic dilemma as many antibiotics disrupt the gut microbiome, including first-line options for rCDI [38]. Although guideline recommended antibiotics can provide some sustained clinical response, they are unable to restore the dysbiosis. The microbiome disruptions caused by antibiotics can then lead to the decreased production of secondary bile acids and SCFAs [20, 39-41], which favors the germination and growth of *C. difficile* [2, 12]. Therefore, treatment methods that can efficiently restore the diversity of the gut microbiome after antibiotics and thereby influence colonization resistance and immune modulation are likely to prevent CDI recurrence.

### Impact of REBYOTA$^{TM}$ (RBL)

The first FDA-approved microbiota-based therapeutic for preventing CDI recurrence, REBYOTA$^{TM}$ (fecal microbiota, live-jslm; 'RBL') (Rebiotix Inc., Roseville, MN) is a commercially prepared, FDA-approved microbiota-based live biotherapeutic. In a pivotal Phase 3 trial (PUNCH CD3, NCT03931941), RBL was evaluated for the prevention of CDI recurrence as a randomized, double-blinded, placebo controlled (2:1) study, in which treatment response was defined as absence of CDI recurrence at 8 weeks after blinded investigational treatment. In that study, RBL met the prespecified

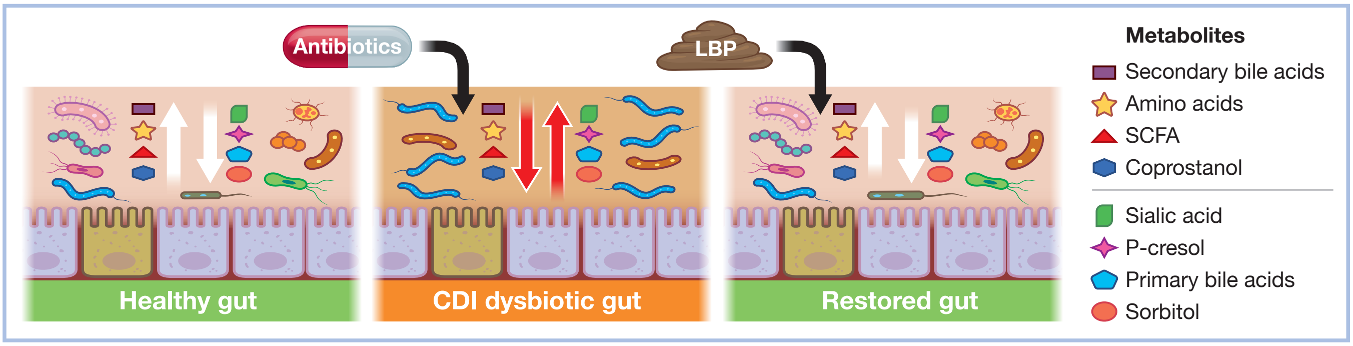

**Permission:** adapted from Aguire A.M., Sorg J.A., Gut Microbes, 2022. 14(1): p. 2094672

**Footnote:** LBP = live biotherapeutic product; SCFA = short-chain fatty acids

### RBL responders have higher secondary bile acids and lower primary bile acids

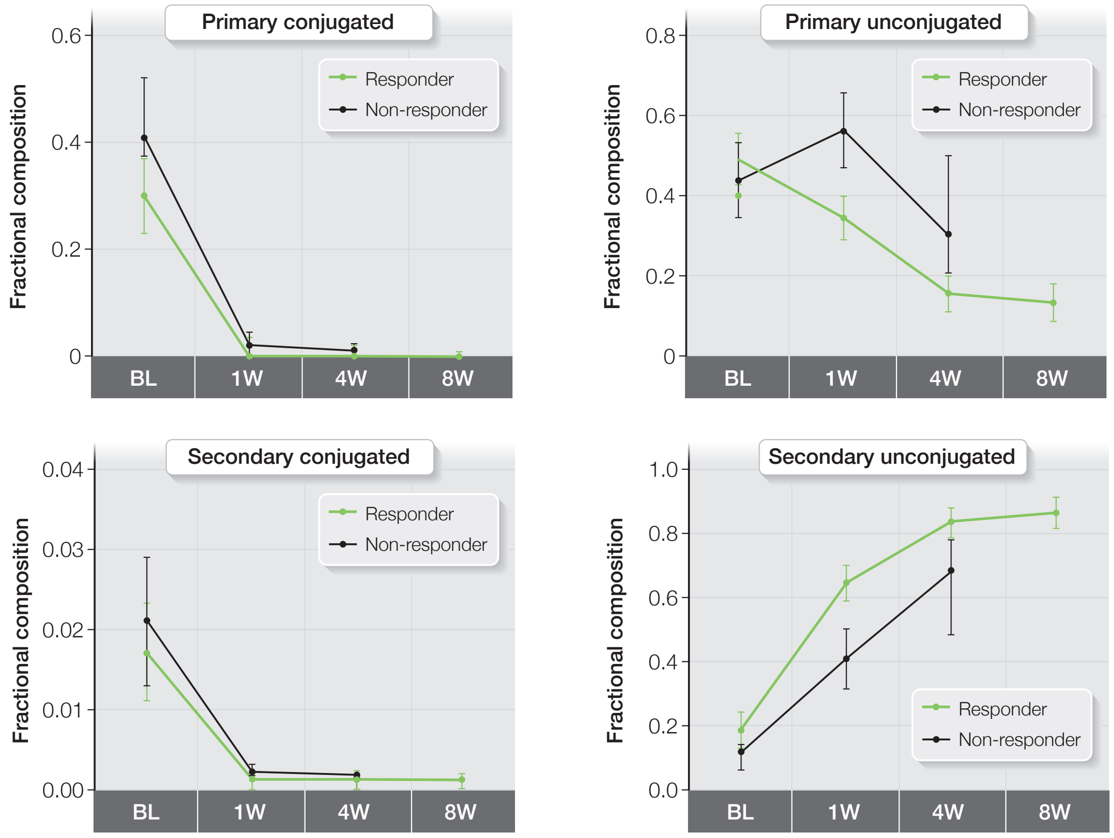

*Data include RBL and placebo treatment groups. Data are represented as mean ± 95% CI*

| Bile acid | Conjugation | ANOVA p-value (rate of change BL to week 1 between groups) |
|---|---|---|
| Primary | Conjugated | 0.075 |
| Primary | Unconjugated | 0.002 |
| Secondary | Conjugated | 0.985 |
| Secondary | Unconjugated | 0.075 |

### RBL leads to significant S:P ratio increase relative to non-responders and placebo-treated responders

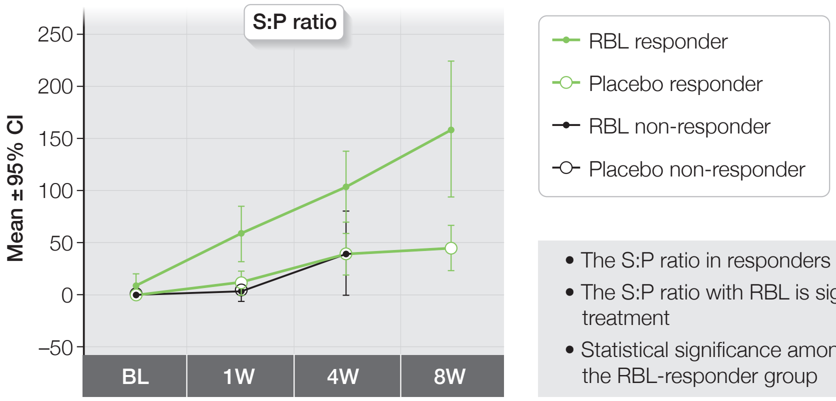

| Predictors | p-value |
|---|---|
| **Outcome** (responder and non-responder) | 0.00033 |
| **Treatment** (RBL and placebo) | 0.02575 |

- The S:P ratio in responders is significantly different than in non-responders
- The S:P ratio with RBL is significantly different than with placebo treatment
- Statistical significance among the various groups was driven by changes in the RBL-responder group

**Footnote:** S = secondary, P = primary, BL = baseline, 1W = 1 week, 4W = 4 week, 8W = 8 week threshold of success, with a 0.986 posterior probability of superiority for RBL vs placebo, thus demonstrating the safety and efficacy of RBL for preventing rCDI [42].

That trial included exploratory analyses of the microbiome composition of administered RBL doses and patient fecal samples from before and after assigned study treatment, as well as liquid chromatography tandem mass spectrometry (LC-MS) analysis of 36 bile acid in patient fecal samples [43].

Those analyses indicated repopulation and restoration of the gut microbiome with Bacteroidetes and Firmicutes [44-47] among patients that responded to RBL. Concurrently, the ratio of secondary to primary (S:P) bile acids was calculated, and it was found that patients responding to RBL had a higher S:P ratio, when compared with pretreatment or placebo, with changes occurring as early as one week post treatment [48]. This shift in bile acid compositions may help suppress spore germination and *C. difficile* toxin production, underpinning the clinical efficacy to prevent CDI recurrence. The bile acid shifts are also consistent with the microbiome changes, in that many of the taxa increased after RBL are known to carry bile acid metabolism genes[48]. SCFA changes with RBL were not evaluated as the principle driving factors behind microbiota restoration were bile acids.

### Conclusion

- The importance of a broad consortium of Bacteroidetes and Firmicutes is manifest in their critical role in colonization resistance and immune modulation [49]
- The essential ability of these taxa to metabolize bile acids and SCFAs confers colonization resistance to various pathogens including *C. difficile*
- Additionally, SCFAs may regulate several leukocyte functions including production of cytokines (TNF-α, IL-2, IL-6 and IL-10), eicosanoids and chemokines
- Restoration of the normal gut microbiome is viewed as very beneficial
- RBL has been shown to restore the essential metabolic processes for a healthy microbiome and avoid recurrent CDI

Corresponding author name: **Dr. Teena Chopra** Detroit Medical Center and Vibra Hospital, Detroit MI – tchopra@med.wayne.edu

InSight title: **Role of the microbiome in essential metabolism in the human gut and its implications for *Clostridioides difficile* infection**

Sponsor: This InSight was sponsored by **Ferring Pharmaceuticals.**

DOI: https://10.1093/ofid/ofad447

OXFORD UNIVERSITY PRESS

Open Forum Infectious Diseases

© The Author(s) 2023. Published by Oxford University Press for the Infectious Diseases Society of America. This is an Open Access article distributed under the terms of the Creative Commons Attribution License (https://creativecommons.org/licenses/by/4.0/), which permits unrestricted reuse, distribution, and reproduction in any medium, provided the original work is properly cited.

### References

1. Marchesi, J.R. and J. Ravel, *The vocabulary of microbiome research: a proposal.* Microbiome, 2015. **3**: p. 31.
2. Seekatz, A.M., N. Safdar, and S. Khanna, *The role of the gut microbiome in colonization resistance and recurrent Clostridioides difficile infection*. Therap Adv Gastroenterol, 2022. **15**: p. 17562848221134396.
3. Khan, I., et al., Mechanism of the Gut Microbiota Colonization Resistance and Enteric Pathogen Infection. Front Cell Infect Microbiol, 2021. 11: p. 716299.
4. Shahinas, D., et al., *Toward an understanding of changes in diversity associated with fecal microbiome transplantation based on 16S rRNA gene deep sequencing*. mBio, 2012. **3**(5).
5. Chang, J.Y., et al., *Decreased diversity of the fecal Microbiome in recurrent Clostridium difficile-associated diarrhea.* J Infect Dis, 2008. **197**(3): p. 435-8.
6. Seekatz, A.M., et al., *Dynamics of the fecal microbiome in patients with recurrent and nonrecurrent Clostridium difficile infection*. Genome Med, 2016. **8**(1): p. 47.
7. Schubert, A.M., et al., *Microbiome data distinguish patients with Clostridium difficile infection and non-C. difficile-associated diarrhea from healthy controls.* mBio, 2014. **5**(3): p. e01021-14.
8. Berkell, M., et al., *Microbiota-based markers predictive of development of Clostridioides difficile infection*. Nat Commun, 2021. **12**(1): p. 2241.
9. Antharam, V.C., et al., *Intestinal dysbiosis and depletion of butyrogenic bacteria in Clostridium difficile infection and nosocomial diarrhea.* J Clin Microbiol, 2013. **51**(9): p. 2884-92.
10. Manges, A.R., et al., *Comparative metagenomic study of alterations to the intestinal microbiota and risk of nosocomial Clostridum difficile-associated disease.* J Infect Dis, 2010. **202**(12): p. 1877-84.
11. Rea, M.C., et al., *Clostridium difficile carriage in elderly subjects and associated changes in the intestinal microbiota*. J Clin Microbiol, 2012. **50**(3): p. 867-75.
12. Alyousef, A.A., *Clostridium difficile: Epidemiology, Pathogenicity, and an Update on the Limitations of and Challenges in Its Diagnosis.* J AOAC Int, 2018. **101**(4): p. 1119-1126.
13. Abad, C.L.R. and N. Safdar, *A Review of Clostridioides difficile Infection and Antibiotic-Associated Diarrhea*. Gastroenterol Clin North Am, 2021. **50**(2): p. 323-340.
14. Martinez-Melendez, A., et al., *An Update on Clostridioides difficile Binary Toxin*. Toxins (Basel), 2022. **14**(5).
15. Vıtek, L. and M. Haluzık, *The role of bile acids in metabolic regulation.* J. Endocrinol, (2016) **228**(3): p. R85–96.
16. Ducarmon, Q.R., et al., *Gut Microbiota and Colonization Resistance against Bacterial Enteric Infection*. Microbiol Mol Biol Rev, 2019. **83**(3).

17. Aguirre, A.M. and J.A. Sorg, *Gut associated metabolites and their roles in Clostridioides difficile pathogenesis*. Gut Microbes, 2022. **14**(1): p. 2094672.

18. Thanissery, R., J.A. Winston, and C.M. Theriot, *Inhibition of spore germination, growth, and toxin activity of clinically relevant C. difficile strains by gut microbiota derived secondary bile acids*. Anaerobe, 2017. **45**: p. 86-100.

19. Sorg, J.A. and A.L. Sonenshein, *Bile salts and glycine as cogerminants for Clostridium difficile spores*. J Bacteriol, 2008. **190**(7): p. 2505-12.

20. Theriot, C.M., et al., *Antibiotic-induced shifts in the mouse gut microbiome and metabolome increase susceptibility to Clostridium difficile infection*. Nat Commun, 2014. **5**: p. 3114.

21. Sorg, J.A. and A.L. Sonenshein, *Inhibiting the initiation of Clostridium difficile spore germination using analogs of chenodeoxycholic acid, a bile acid*. J Bacteriol, 2010. **192**(19): p. 4983-90.

22. Sorg, J.A. and A.L. Sonenshein, *Chenodeoxycholate is an inhibitor of Clostridium difficile spore germination*. J Bacteriol, 2009. **191**(3): p. 1115-7.

23. Allegretti, J.R., et al., *Recurrent Clostridium difficile infection associates with distinct bile acid and microbiome profiles*. Aliment Pharmacol Ther, 2016. **43**(11): p. 1142-53.

24. Buffie, C.G., et al., *Precision microbiome reconstitution restores bile acid mediated resistance to Clostridium difficile*. Nature, 2015. **517**(7533): p. 205-8.

25. Theriot, C.M., A.A. Bowman, and V.B. Young, *Antibiotic-Induced Alterations of the Gut Microbiota Alter Secondary Bile Acid Production and Allow for Clostridium difficile Spore Germination and Outgrowth in the Large Intestine*. mSphere, 2016. **1**(1).

26. Hayashi, A., et al., *The Butyrate-Producing Bacterium Clostridium butyricum Suppresses Clostridioides difficile Infection via Neutrophil- and Antimicrobial Cytokine-Dependent but GPR43/109a-Independent Mechanisms*. J Immunol, 2021. **206**(7): p. 1576-1585.

27. Hryckowian, A.J., et al., *Microbiota-accessible carbohydrates suppress Clostridium difficile infection in a murine model*. Nat Microbiol, 2018. **3**(6): p. 662-669.

28. Martin-Gallausiaux, C., et al., *SCFA: mechanisms and functional importance in the gut*. Proc Nutr Soc, 2021. **80**(1): p. 37-49.

29. Fachi, J.L., et al., *Butyrate Protects Mice from Clostridium difficile-Induced Colitis through an HIF-1-Dependent Mechanism*. Cell Rep, 2019. **27**(3): p. 750-761 e7.

30. Ouyang, Z.R., et al., *The role of short-chain fatty acids in Clostridioides difficile infection*: A review. Anaerobe, 2022. **75**: p. 102585.

31. Fachi, J.L., et al., *Acetate coordinates neutrophil and ILC3 responses against C. difficile through FFAR2*. J Exp Med, 2020. **217**(3).

32. Nurmi, J.T., P.A. Puolakkainen, and N.E. Rautonen, *Bifidobacterium Lactis sp. 420 up-regulates cyclooxygenase (Cox)-1 and down-regulates Cox-2 gene expression in a Caco-2 cell culture model*. Nutr Cancer, 2005. **51**(1): p. 83-92.

33. Tong, L.C., et al., *Propionate Ameliorates Dextran Sodium Sulfate-Induced Colitis by Improving Intestinal Barrier Function and Reducing Inflammation and Oxidative Stress*. Front Pharmacol, 2016. **7**: p. 253.

34. Tan, J., et al., *The role of short-chain fatty acids in health and disease*. Adv Immunol, 2014. **121**: p. 91-119.

35. Rivera-Chavez, F., et al., *Depletion of Butyrate-Producing Clostridia from the Gut Microbiota Drives an Aerobic Luminal Expansion of Salmonella*. Cell Host Microbe, 2016. **19**(4): p. 443-54.

36. Vigsnaes, L.K., et al., 2'FL and LNnT Exert Antipathogenic Effects against C. difficile ATCC 9689 In Vitro, Coinciding with Increased Levels of Bifidobacteriaceae and/or Secondary Bile Acids. Pathogens, 2021. 10(8).

37. McDonald, L.C., et al., *Clinical Practice Guidelines for Clostridium difficile Infection in Adults and Children: 2017 Update by the Infectious Diseases Society of America (IDSA) and Society for Healthcare Epidemiology of America (SHEA)*. Clin Infect Dis, 2018. **66**(7): p. e1-e48.

38. Tannock, G.W., et al., *A new macrocyclic antibiotic, fidaxomicin (OPT-80), causes less alteration to the bowel microbiota of Clostridium difficile-infected patients than does vancomycin*. Microbiology (Reading), 2010. **156**(Pt 11): p. 3354-3359.

39. Hoverstad, T., et al., *Influence of ampicillin, clindamycin, and metronidazole on faecal excretion of short-chain fatty acids in healthy subjects*. Scand J Gastroenterol, 1986. **21**(5): p. 621-6.

40. McDonald, J.A.K., et al., *Inhibiting Growth of Clostridioides difficile by Restoring Valerate, Produced by the Intestinal Microbiota*. Gastroenterology, 2018. **155**(5): p. 1495-1507 e15.

41. Ferreyra, J.A., et al., *Gut microbiota-produced succinate promotes C. difficile infection after antibiotic treatment or motility disturbance*. Cell Host Microbe, 2014. **16**(6): p. 770-7.

42 Khanna, S., et al., *Efficacy and Safety of RBX2660 in PUNCH CD3, a Phase III, Randomized, Double-Blind, Placebo-Controlled Trial with a Bayesian Primary Analysis for the Prevention of Recurrent Clostridioides difficile Infection*. Drugs, 2022.

43. Papazyan, R., et al., *Human Fecal Bile Acid Analysis after Investigational Microbiota-Based Live Biotherapeutic Delivery for Recurrent Clostridioides difficile Infection*. Microorganisms, 2023. **11**(1): p. 135.

44. Blount, K., et al., *Treatment success in reducing recurrent Clostridioides difficile infection with investigational live biotherapeutic RBX2660 is associated with microbiota restoration: Consistent evidence from a Phase 3 clinical trial*. Open Forum Infect Dis, 2021. **8**: p. S624-S625.

45. Blount, K.F., et al., *Restoration of Bacterial Microbiome Composition and Diversity Among Treatment Responders in a Phase 2 Trial of RBX2660: An Investigational Microbiome Restoration Therapeutic*. Open Forum Infect Dis, 2019. **6**(4): p. ofz095.

46. Orenstein, R., et al., *Durable reduction of Clostridioides difficile infection recurrence and microbiome restoration after treatment with RBX2660: results from an open-label phase 2 clinical trial*. BMC Infect Dis, 2022. **22**(1): p. 245.

47. Kwak, S., et al., *Impact of investigational microbiota therapeutic RBX2660 on the gut microbiome and resistome revealed by a placebo-controlled clinical trial*. Microbiome, 2020. **8**(1): p. 125.

48. Papazyan, R., et al., *Rapid restoration of bile acid compositions after treatment with RBX2660 for recurrent Clostridioides difficile infection: results from the PUNCH CD3 phase 3 trial [poster], in In: ID Week*; September 29 - October 3. 2021: Virtual.

49. Pickard, J.M., et al., *Gut microbiota: Role in pathogen colonization, immune responses, and inflammatory disease*. Immunol Rev, 2017. **279**(1): p. 70-89.

**Acknowledgment,** the authors thank Angela Donald ND for writing support.

### Conflict of Interest Statement:

**Teena Chopra** is a speaker for Ferring Pharmaceuticals, **Phil Hylemon** is supported by Veterans Administration BRAVE funds but no conflict to declare, **Glenn Tillotson** is a consultant to Ferring Pharmaceuticals and Spero Therapeutics.
